# Announcement

**Published:** 1895-06

**Authors:** 


### Editorial.

### ANNOUNCEMENT.

With the July issue, the SOUTHERN MEDICAL RECORD wil change hands. It will then pass under the ownership and control of Dr. Bernard Wolff, who will conduct it in the future. The present efficient corps of collaborators will be retained and will lend their aid toward keeping up the high standard of excellence maintained by it for nearly twenty-six years. The journal will be enlarged from time to time by addition of departments devoted to the several provinces of medicine and by special features. It will be the earnest endeavor of the new management to make the SOUTHERN MEDICAL RECORD a high-class publication, and a true, accurate and instructive exponent of current medical thought and progress. We trust that our friends will lend us their assistance and co-operation in carrying out this praiseworthy intention. Contributions of merit are solicited from all quarters. Address all communications in the future to

BERNARD WOLFF, M. D., Editor and Proprietor,
Box 436. SOUTHERN MEDICAL RECORD, Atlanta, Ga.

---

Not long since at a public gathering, one of Georgia's most distinguished lawyers made the remark that it would be a sad day for the public and the medical profession, when physicians ceased to uphold and live up to the code of medical ethics, a code which inculcated principles and practices as elevating and as ennobling as weie ever formulated as a guide for the conduct of any body of men, social or religious.

Such an utterance did credit to the wisdom and honesty of the man who made it: let us see then that we live up to this high standard, not only in letter, but in spirit.

At the last meeting of the State Medical Association, one of its members was expelled for violation of the code by advertising in the daily press.

To such action of the association we believe there was not a dissenting voice, yet the daily press affords rather frequent instances of similar violation, in a lesser degree, by members of the profession in good standing. Is such a state of affairs just?—is it fair? When a physician leaves home for any length of time, a simple announcement by a paper of his departure or return, is a legitimate item of news and affords information to the patients (and creditors) of the physician, but when such notice is repeated frequently and at intervals and is accompanied by a fulsome account of the physician's ability and renown, has not the code been violated; and, if such notices have been made without the knowledge of the physician by some misguided but well-meaning friend, does it not become the duty of the physician to prevent its repetition rather than to furnish data for another occasion?

Let us not in the desire to get the almighty dollar seek a short cut to distinction, but rather let is by honesty and diligence climb more slowly the ladder of fame, ever keeping before us the high and lofty aims of our life work, and though we die without having acquired wealth or achieved distinction we shall have possessed that greater reward, a conscience void of offense.

